# Prediction of Resectability of Peritoneal Disease in Ovarian Cancer Patients Using the Peritoneal Cancer Index (PCI) and Fagotti Score on MRI

**DOI:** 10.3390/cancers18010165

**Published:** 2026-01-02

**Authors:** Marianna Konidari, Sofia Gourtsoyianni, Nikolaos Thomakos, Georgia Lymperopoulou, Chara Tzavara, Vasilios Pergialiotis, Alexandros Rodolakis, Lia Angela Moulopoulos, Charis Bourgioti

**Affiliations:** 1 1st Department of Radiology, School of Medicine, National and Kapodistrian University of Athens, Areteion Hospital, 11528 Athens, Greece; 2 1st Department of Obstetrics and Gynecology, School of Medicine, National and Kapodistrian University of Athens, Alexandra Hospital, 11528 Athens, Greece; 3 Biostatistics Department, National and Kapodistrian University of Athens, 15784 Athens, Greece

**Keywords:** ovarian cancer, MRI, peritoneal carcinomatosis, cytoreduction, Peritoneal Cancer Index, Fagotti score, Diffusion-Weighted Imaging

## Abstract

Background/Objectives: Cytoreduction status is a critical prognostic factor in ovarian cancer, yet preoperative selection of patients suitable for primary debulking surgery and accurate prediction of surgical outcome remain challenging. This study aimed to evaluate the prognostic ability of MRI-based Fagotti score and Peritoneal Cancer Index (PCI) for predicting resectability of peritoneal disease in ovarian cancer patients. Methods: This was a prospective single-center observational study. Patients with suspected primary ovarian cancer who underwent preoperative MRI of the abdomen and pelvis with a dedicated protocol were considered. MRI-based Fagotti score and PCI were determined by two readers independently, using a combination of T2W, Diffusion-Weighted Imaging (DWI), and contrast-enhanced T1W sequences. In cases of discordance, a third radiologist reviewed the scans and consensus was reached. ROC analysis and logistic regression were used to evaluate prognostic performance. The reference standard to predict resectability was optimal cytoreduction defined as residual disease ≤1 cm. Results: Forty-six women with epithelial ovarian cancer (mean age 56.3 ± 2.6 years) who underwent preoperative MRI, followed by laparoscopy and/or laparotomy, were included in the study. Both MRI-based Fagotti score and PCI showed high predictive value for predicting resectability (AUC 0.92 and 0.94, respectively). Optimal cut-offs were ≤6 for Fagotti score and ≤20 for PCI. Patients with scores below these thresholds had >60-fold (Fagotti) and >100-fold (PCI) increased odds for successful primary cytoreduction (*p* < 0.001). Conclusions: MRI-based Fagotti score and PCI may serve as powerful noninvasive predictors of surgical outcome in ovarian cancer. MRI may reliably guide treatment decisions, reducing unnecessary laparotomies and optimizing patient selection.

## Simple Summary

Optimal cytoreduction is the strongest prognostic factor in ovarian cancer presenting with peritoneal disease. However, selecting patients for primary cytoreductive surgery versus neoadjuvant chemotherapy remains challenging. In this study, we evaluated the prognostic performance of MRI-based Fagotti score and Peritoneal Cancer Index (PCI) in women with ovarian cancer. Both MRI-based Fagotti score and PCI demonstrated high accuracy for predicting surgical feasibility, with optimal cut-offs (Fagotti ≤ 6, PCI ≤ 20) strongly associated with optimal cytoreduction. These findings suggest that MRI-based scores can serve as an alternative noninvasive tool for guiding treatment decisions and surgical planning in ovarian cancer patients.

## 1. Introduction

Epithelial ovarian cancer is the most fatal gynecologic malignancy, with a 5-year survival rate of approximately 30–40%, largely due to its late-stage presentation and extensive peritoneal dissemination at diagnosis. The primary therapeutic approach for advanced ovarian cancer is primary debulking surgery (PDS), with the objective of achieving maximal tumor resection [1]. In cases where complete resection is not technically feasible due to extent or distribution of disease (non-resectable disease), or when patients are considered unfit for extensive surgery (inoperability), neoadjuvant chemotherapy followed by interval debulking surgery (IDS) constitutes an alternative treatment [2,3,4,5,6,7]. The strongest independent prognostic factor for survival in advanced disease is the extent of cytoreduction achieved, with complete macroscopic resection conferring the greatest benefit [8,9,10,11]. However, the choice between primary debulking surgery and neoadjuvant chemotherapy remains complex, and unnecessary laparotomies performed in patients unlikely to achieve optimal resection are associated with significant morbidity and delayed systemic therapy.

Preoperative estimation of tumor load and disease at critical sites is a major factor in the treatment decision-making process in ovarian cancer surgery. Surgical predictive scores, such as the Fagotti score on laparoscopy and the Peritoneal Cancer Index (PCI) on laparotomy, improve prediction but remain invasive [12,13,14]. Contrast-enhanced computed tomography (CT), Magnetic Resonance Imaging (MRI), and Positron Emission Tomography—Computed Tomography (PET-CT) are other options for the preoperative evaluation of patients with ovarian cancer [15,16]. CT is the most accessible and commonly used imaging modality for initial assessment; however, it has limited accuracy in predicting surgical outcomes [15,16]. MRI, particularly with Diffusion-Weighted Imaging (DWI), has emerged as a promising and accurate noninvasive modality for assessing the extent and predicting non-resectability of peritoneal carcinomatosis [16,17].

The Fagotti score and PCI are validated intraoperative tools. A Fagotti score ≥ 8 can predict residual disease with an overall accuracy of 75%, a positive predictive value of 100% and a negative predictive value of 70% [12]. Several studies have confirmed the prognostic ability of PCI in ovarian cancer, demonstrating a strong association between intraoperative peritoneal assessment, surgical outcomes, and patient prognosis [18,19,20,21]. However, the role of an MRI-based Fagotti score or PCI requires further investigation. Few studies have directly compared MRI-based scores against surgical outcomes, and optimal cut-off values remain under investigation [22,23,24,25].

The aim of this study was to evaluate the prognostic value of MRI-based Fagotti score and PCI in predicting feasibility of primary cytoreduction and surgical outcome in ovarian cancer patients.

## 2. Materials and Methods

### 2.1. Study Population

This was a prospective single-center observational cohort study. The study protocol was approved by the institutional ethics review board (315/26-03-2021). Patients with suspected primary ovarian cancer at initial investigation between April 2021 and April 2025 were enrolled and referred for preoperative MRI of the abdomen and pelvis. All patients were considered potential candidates for cytoreductive surgery and scheduled for diagnostic laparoscopy and/or exploratory laparotomy within a four-week time interval from the MRI scan.

Exclusion criteria were: (1) contraindications to MRI (e.g., pacemakers, metallic foreign bodies), (2) patients unfit for surgery, (3) time lapse between imaging and surgery > 4 weeks, (4) biopsy-proven non-epithelial ovarian tumors, benign or borderline ovarian tumors, non-primary ovarian cancer, (5) informed consent not provided.

### 2.2. MRI Protocol

All MRI scans of the abdomen and pelvis were performed on a 3.0 Tesla MRI unit (Ingenia, Philips Healthcare, NL) using an external multichannel array body surface coil. The dedicated MRI protocol consisted of axial T2W sequences (with and without fat saturation, slice thickness 5 mm), axial DWI sequences (b = 0 and b = 1200 s/mm^2^), and T1W sequences in axial/coronal view after gadolinium administration (gadoterate meglumine, 0.5 mmol/mL). Axial pre-contrast T1W and coronal T2W sequences were also included. Apparent Diffusion Coefficient (ADC) maps were extracted from the DWI sequence. An antiperistaltic agent (butylscopolamine bromide, 20 mg) was administered intramuscularly before the examination. Total scan time was 50–60 min. Technical details of the applied MRI protocol can be found in Table S1.

### 2.3. Radiological Evaluation

Two experienced radiologists with 5 and 15 years of experience in gynecological oncologic imaging evaluated the preoperative MRI scans independently. All clinical and laboratory data of the initial preoperative investigation were available during radiological evaluation.

**Figure 1:**
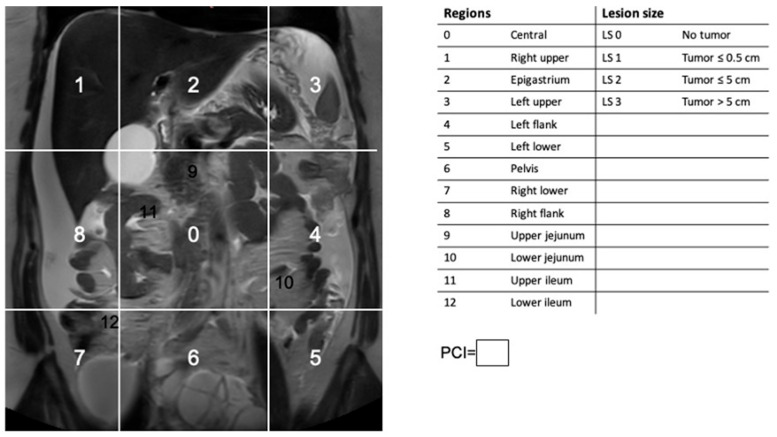
MRI-based Peritoneal Cancer Index (PCI) adapted from the laparotomic score used to determine peritoneal tumor load. The overall PCI score is obtained by adding all individual regional scores, yielding a range from 0 to 39.

The MRI-based Fagotti score and PCI were adapted from the laparoscopic and surgical scores, respectively (Table 1, Figure 1) [12,13]. All seven sites of the Fagotti score and thirteen anatomical regions of the PCI were evaluated in all available MRI sequences in combination for the presence and size of peritoneal implants. On MRI, peritoneal deposits appear as solid soft tissue nodules, plaques, masses or confluent disease (i.e., omental cake) that demonstrate intermediate signal intensity on T2W sequences, restricted diffusion indicated by high signal on the high b value DWI images and contrast enhancement on the T1W sequence after gadolinium administration. Imaging features of gastrointestinal (bowel or stomach) involvement include nodules, a well-defined mass, segmental wall thickening or diffuse serosal infiltration. Mesenteric involvement, apart from nodules or masses of varying size, may be indicated by secondary signs, including mesenteric retraction or tethering and angulation or kinking of bowel loops.

The Fagotti score, a laparoscopic scoring system developed to predict the likelihood of achieving optimal cytoreduction (≤1 cm residual disease) in advanced ovarian cancer, focuses on disease at seven individual sites as evaluated during diagnostic laparoscopy: omentum, peritoneum, diaphragm, mesentery, bowel, stomach and liver surface. Each site receives a score 0 (no disease) or 2 (disease is present). The sum of all scores yields the Predictive Index Value (PIV), which ranges from 0 to 14 (Table 1). A PIV ≥ 8 predicts suboptimal cytoreduction (residual disease > 1 cm) with high accuracy. Patients with PIV ≥ 8 are usually directed toward neoadjuvant chemotherapy instead of PDS [12].

The Peritoneal Cancer Index (PCI) divides the peritoneal cavity and intraperitoneal organs into 13 distinct regions, defined by anatomical landmarks of the abdominal and pelvic compartments [13]. The peritoneal cavity is segmented into nine abdominopelvic areas using two vertical midclavicular lines and two transverse lines—one at the level of the anterior superior iliac spines and another below the costal margins. These regions are numbered 0–8 in a clockwise sequence, beginning at the umbilical area. The small bowel is assessed separately and subdivided into four regions, designated as regions 9–12. Within each region, tumor burden is graded using a four-point scale: 0, no visible disease; 1, tumor nodules <0.5 cm (minimal disease); 2, nodules 0.5–5 cm (moderate disease); and 3, nodules >5 cm or confluent disease. The overall PCI score is obtained by adding all individual regional scores, yielding a range from 0 to 39 (Figure 1). Higher PCI values indicate greater tumor burden and lower likelihood of optimal cytoreduction [18].

Each of the seven disease sites of the Fagotti score and the thirteen regions of the PCI were evaluated by each reader on T2W, DWI, and T1W post-contrast sequences in combination to calculate the MRI-based scores. In cases of discordant scoring between the two primary readers, a third senior radiologist with 25 years of experience reviewed the scans. The third reader was blinded to surgical outcome and resolved discrepancies by consensus in order to provide a final score.

**Figure 2:**
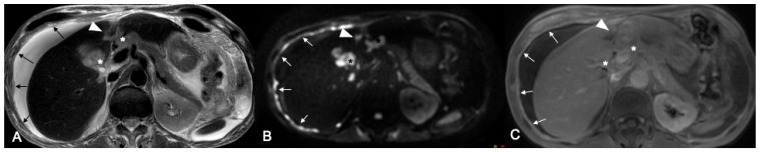
Axial MR images of the upper abdomen. (**A**) T2W image shows ill-defined soft tissue at the porta hepatis and perihilar region (asterisks) and along the falciform ligament (arrowhead). Widespread infiltration of the right hemidiaphragm is also present (arrows). (**B**) Peritoneal deposits demonstrate high signal intensity on the b 1000 DWI image, indicative of restricted diffusion. (**C**) On the T1W post-contrast sequence with fat saturation, peritoneal deposits show contrast enhancement. Diffuse enhancement of the right hemidiaphragm also noted.

**Figure 3:**
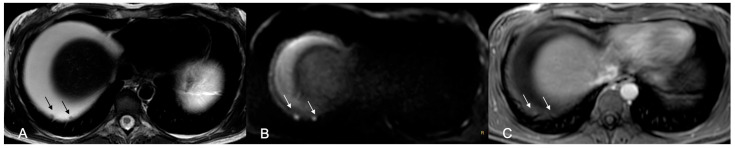
Axial MR images at the level of the liver dome. T2W (**A**), DWI b 1000 (**B**), and T1W post-contrast with fat saturation (**C**) images demonstrate two small up to 5 mm solid nodules on the right hemidiaphragm (arrows in (**A**)), with restricted diffusion (**B**) and contrast enhancement (**C**).

**Figure 4:**
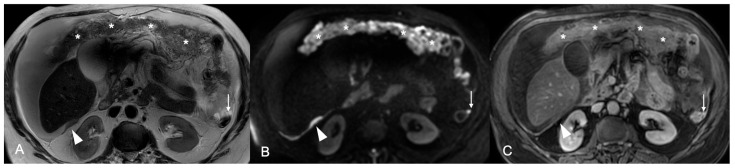
Axial MR images of the upper abdomen. (**A**) T2W image shows diffuse involvement of the greater omentum with “omental cake” pattern (asterisks). There is also a 15 mm nodule in the Morisson space (arrowhead) and a 5 mm nodule in the left paracolic gutter (arrow). Peritoneal deposits demonstrate high signal intensity on the b 1000 DWI image (**B**), indicative of restricted diffusion, and enhancement on the T1W post-contrast sequence with fat saturation (**C**).

**Figure 5:**
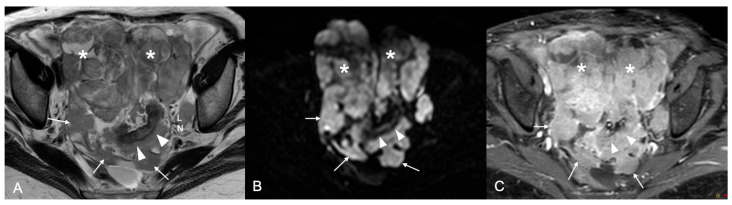
Axial MR images of the pelvis. (**A**) T2W image shows large, confluent mixed cystic-solid ovarian masses (asterisks) along with diffuse peritoneal involvement of the pelvic peritoneum (arrows) and large deposits on the sigmoid bowel serosa (arrowheads). Enlarged left internal iliac node also present (LN). The ovarian as well as the peritoneal masses demonstrate high signal intensity on the b 1200 DWI image (**B**), indicative of restricted diffusion, and intense enhancement on the T1W sequence post-contrast with fat saturation (**C**).

**Figure 6:**
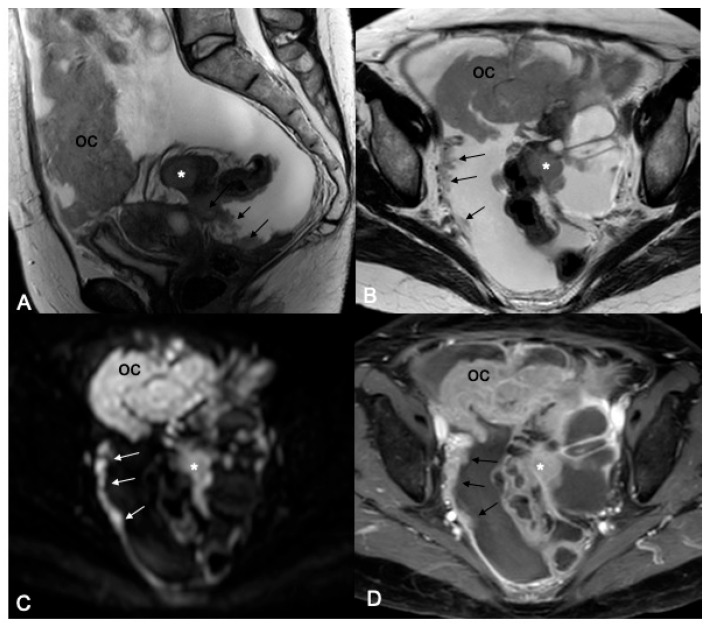
Sagittal (**A**) and axial (**B**–**D**) MR images of the pelvis. Sagittal (**A**) and axial (**B**) T2W images show a 3 cm peritoneal deposit infiltrating the sigmoid colon (asterisk). There is also involvement of the pelvic peritoneum and Douglas pouch (arrows). “Omental cake” also present (oc). Peritoneal disease demonstrates high signal intensity on the b 1200 DWI image (**C**), indicative of restricted diffusion, and enhancement on the T1W post-contrast sequence with fat saturation (**D**).

Figure 2, Figure 3, Figure 4, Figure 5 and Figure 6 show examples of peritoneal disease as seen on T2W, DWI, and T1W post-contrast sequences (Figure 2, Figure 3, Figure 4, Figure 5 and Figure 6).

### 2.4. Reference Standard

Intraoperative findings and surgical outcome were the reference standard for prediction of resectability. Treatment decision between primary or interval debulking surgery was based on departmental guidelines, taking into account patient comorbidities and disease-related factors. Primary cytoreductive surgery was performed if considered feasible on initial exploration with laparoscopy and/or laparotomy. After maximum effort to remove all visible disease, surgeons described the surgical outcome as: complete (no macroscopic residual tumor); optimal (≤1 cm residual tumor); suboptimal (>1 cm residual tumor); non-feasible (as determined by initial exploration). Resectable disease included cases with complete or optimal cytoreduction. Non-resectable disease included cases with suboptimal cytoreduction and cases with non-feasible cytoreduction [7].

### 2.5. Statistical Analysis

Quantitative variables were expressed as mean values (±SD) and as median (IQR), while categorical variables were described as absolute and relative frequencies. Quantitative variables were tested for normality using the Kolmogorov–Smirnov criterion. ROC curves were used to estimate the predictive ability of MRI for resectability of peritoneal disease. The area under the curve (AUC) was also calculated. Optimal cut-offs were defined by Youden’s index, with corresponding sensitivity, specificity, PPV, and NPV. Logistic regression models were used with independent variables the MRI scores, according to the cut-offs that emerged from ROC analysis, to calculate odds ratios (ORs). Interobserver agreement was evaluated using Intraclass Correlation Coefficient (ICC). ICC < 0.5 indicated poor reliability, ICC 0.5–0.75 moderate reliability, ICC 0.75–0.9 good reliability and ICC > 0.9 excellent reliability. All reported *p*-values were two-tailed. Statistical significance was set at *p* < 0.05. Analyses were conducted using SPSS v27.

## 3. Results

### 3.1. Study Group

Forty-six of the 80 patients initially recruited to the study were included in the final analysis. A total of 34 patients were excluded, 30 because they were diagnosed with other than epithelial ovarian cancer tumors (4/30 non-epithelial ovarian cancer; 16/30 benign or borderline tumors; 10/30 non-ovarian tumors) and four because they were unable to complete the MRI exam (e.g., claustrophobia). Clinical, surgical and histological characteristics of the study population are reported in Table 2. Patients’ mean age was 56.3 (SD = 2.6) years. Forty out of the forty-six (87%) patients had advanced (III-IV) FIGO stage. A total of 27/46 patients (58.7%) underwent primary debulking surgery with complete or optimal cytoreduction. Mean MRI-based Fagotti score was 6 (SD = 3.8) and mean MRI-based PCI was 16.7 (SD = 7.8).

### 3.2. MRI-Based Fagotti Score

**Figure 7:**
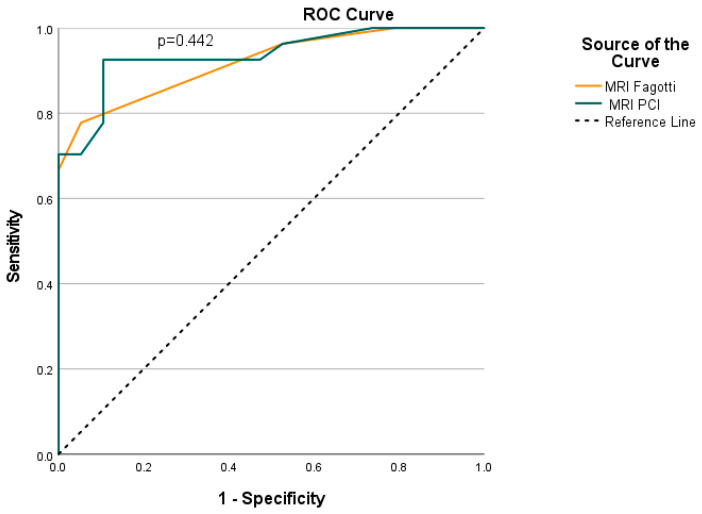
ROC curves for MRI-based Fagotti score and PCI for predicting optimal cytoreduction.

MRI-based Fagotti score had significant prognostic ability for predicting resectable disease, with an AUC of 0.92 (95% CI: 0.84–1.00; *p* < 0.001) (Table 3). Optimal cut-off was 6, with sensitivity 77.8%, specificity 94.7%, PPV 95.5% and NPV 75% (Figure 7). Patients with MRI-based Fagotti score ≤6 had 63 times greater probability of optimal resection than patients with MRI-based Fagotti score > 6 (OR = 63; 95% CI: 6.9–573.5; *p* < 0.001).

### 3.3. MRI-Based PCI

MRI-based PCI had significant prognostic ability for predicting resectable disease, with an AUC of 0.94 (95% CI: 0.87–1.00; *p* < 0.001) (Table 3). Optimal cut-off was 20, with sensitivity 92.6%, specificity 89.5%, PPV 92.6% and NPV 89.5% (Figure 7). Patients with MRI-PCI ≤ 20 had 106.3 times greater probability of optimal cytoreduction, than patients with MRI-PCI > 20 (OR = 106.3; 95% CI: 13.6–829.1; *p* < 0.001).

### 3.4. MRI-Based Fagotti Score vs. MRI-Based PCI

Both MRI-based Fagotti score and PCI had significant prognostic ability for predicting resectable disease, without significant differences (*p* = 0.442), indicating similar prognostic ability (Figure 7).

### 3.5. Interobserver Variability

Interobserver agreement was good to excellent for both MRI-based Fagotti score (ICC = 0.91, *p* < 0.001) and MRI-based PCI (ICC = 0.88, *p* < 0.001).

## 4. Discussion

Accurate preoperative prediction of cytoreductive feasibility remains a critical challenge in the management of ovarian cancer. This prospective single-center study demonstrated that both MRI-based Fagotti score and MRI-based PCI were accurate predictors of optimal cytoreduction in ovarian cancer patients. The two MRI-based scores were equally accurate in identifying patients with resectable disease, demonstrating AUCs of 0.92 and 0.94 for MRI-based Fagotti score and MRI-based PCI, respectively, and high odds ratios for optimal cytoreduction. Using the MRI-derived cut-off values of Fagotti ≤ 6 and PCI ≤ 20, both scoring systems achieved high discriminatory performance, supporting their role as potential clinically useful triage tools.

Current evidence indicates that maximal effort primary debulking surgery performed in specialized gynecologic oncology centers, followed by platinum-based chemotherapy and, when appropriate, maintenance therapy, provides the most favorable outcomes with acceptable morbidity [1,14,15]. Among prognostic factors, the amount of residual disease is considered the most significant determinant of survival. In a pooled analysis of three multicenter phase III trials (AGO-OVAR 3, 5, and 7), 3-year overall survival was 72.4% for patients who underwent complete resection, 65.8% for those with optimal resection, and 45.2% for those with residual disease > 1 cm [8,26].

Laparotomy remains the most accurate way of assessing peritoneal tumor load and predicting surgical outcome. The predictive ability for non-resectability using the PCI is high, with AUCs ranging from 0.69 to 0.94 [14,20,27,28,29]. However, laparotomy is a highly invasive procedure, associated with substantial risk of complications and, importantly, it may delay initiation of chemotherapy in patients with non-resectable disease, adversely impacting survival. To address these limitations, diagnostic laparoscopy was introduced as a less invasive alternative, enabling accurate triage of patients for appropriate treatment planning. Advantages of laparoscopy compared to laparotomy include shorter operating times, fewer complications, faster recovery, and prompt initiation of therapy in cases of unresectable disease. Using a cut-off of 8, Fagotti score AUCs range between 0.66 and 0.98 for predicting optimal cytoreduction [14,28,30,31,32,33,34]. Nonetheless, a major disadvantage of laparoscopy is the limited assessment of certain peritoneal sites, such as the mesenteric root, lesser sac, and gastrosplenic ligament [35].

In recent years, abdominal CT has been recommended as the standard imaging modality for staging ovarian cancer [36]. Several studies have investigated the use of preoperative CT for assessing non-resectability through predictive models. Many studies have validated existing models, such as the PCI, reporting moderate performance, with AUCs of 0.55–0.76 [28,29,37,38]. Although CT is the most rapid and widely available imaging technique, it has limitations in detecting small volume carcinomatosis, especially at the mesentery and bowel serosa. MRI, with its high soft tissue resolution and tumor-sensitive sequences such as DWI, is currently considered more accurate than CT, with increased sensitivity for the assessment of multiple sites of disease in epithelial ovarian cancer [25].

Our findings are consistent with prior studies that have explored MRI-adapted peritoneal scoring systems and the prognostic value of MRI in the prediction of surgical outcome. Engbersen et al. reported high agreement between MRI-based PCI and surgical PCI, with AUCs of 0.96–0.98 for the prediction of complete cytoreduction, similar to those of our study (AUC 0.94) [22]. Moreover, the proposed PCI cut-offs of 20 and 15 for reader 1 and reader 2, respectively, align with our results (PCI ≤ 20). Similarly, the ISAAC (Imaging Study on Advanced ovArian Cancer) multicenter study demonstrated that the radiological PCI obtained with MRI was predictive of non-resectability, with an AUC of 0.8 and cut-off of >12 [23]. While these studies primarily focused on PCI, our work extends the existing literature by prospectively evaluating the two most commonly used scoring systems (PCI and Fagotti score) within the same cohort, allowing direct comparison of their predictive performance under identical imaging and clinical conditions. Our results support the evidence that MRI-based Fagotti score and PCI can be reliably adapted to imaging and may be used as predictors of optimal cytoreduction.

Our MRI-based optimal cut-offs (Fagotti ≤ 6, PCI ≤ 20) were also consistent with thresholds used in the intraoperative setting. Fagotti et al. originally validated a laparoscopic predictive index, showing that scores ≥ 8 were strongly predictive of suboptimal cytoreduction [12]. Regarding PCI, surgical cut-offs vary between different centers; however, in the literature, values of 16 and 20 are suggested to predict complete and optimal resection, respectively, whereas neoadjuvant chemotherapy could be considered if the PCI is >24 [19,29,39]. Our results extend this body of work by directly comparing MRI-based Fagotti score and PCI, showing similar prognostic accuracy with slightly higher discriminatory performance for PCI. This is clinically relevant, as it provides two complementary MRI-based tools to support patients’ stratification and treatment planning.

Furthermore, our study reinforces the growing consensus that MRI with DWI, is an accurate tool for the preoperative assessment of peritoneal carcinomatosis in ovarian cancer [16]. Several studies have shown that MRI outperforms CT in detecting small peritoneal implants and in assessing resectability. Michielsen et al. reported that MRI achieved sensitivities up to 90% in detecting peritoneal disease, significantly higher than CT, confirming its value for preoperative staging [24]. Low at al. showed that MRI more accurately predicted PCI in the preoperative setting compared to CT, with no statistical difference between MRI-based and surgical PCI [40]. Rizzo et al. highlighted the high accuracy of DW-MRI in the assessment of multiple sites of disease associated with suboptimal cytoreduction and its superiority to CT for specific unresectable sites [25]. Although our study was not designed to compare MRI directly with CT, our results confirmed that MRI-based scoring systems provided accurate prediction of surgical outcome. The high accuracy of MRI-based scores for predicting optimal cytoreduction in our study, supports current guideline recommendations that MRI should be integrated into the preoperative assessment of advanced ovarian cancer [15,16].

The novelty of the present study lies in demonstrating that MRI-based Fagotti score and PCI yield comparable and reproducible predictive accuracy when applied prospectively using a standardized MRI protocol. Furthermore, we propose MRI-based cut-off values, which are consistent with thresholds previously reported for surgical and radiological assessment. Our study strengthens the growing evidence that MRI-based Fagotti score and PCI offer robust, noninvasive alternatives to surgical procedures for assessing cytoreduction feasibility in ovarian cancer and selecting patients most likely to benefit from primary debulking surgery. Patients with MRI-based PCI > 20 or Fagotti score > 6 are unlikely to achieve optimal cytoreduction, and may rather benefit from neoadjuvant chemotherapy, thus avoiding unnecessary surgery and delays in treatment initiation.

Strengths of this study include its prospective design, use of validated surgical endpoints, and detailed imaging evaluation across multiple MRI sequences.

Limitations include the relatively small, single-center cohort, and radiological evaluation performed by experienced radiologists in gynecological imaging, which may limit external generalizability. The high odds ratios observed in our analysis reflect strong separation between resectable and non-resectable disease; however, we acknowledge that the limited sample size and wide confidence intervals raise the possibility of model overfitting. For this reason, predictive performance should be interpreted with caution, primarily through AUC values and clinical discrimination rather than absolute odds ratio magnitude. Larger multicenter studies will be needed to validate the proposed cut-offs and assess reproducibility across readers and institutions.

## 5. Conclusions

This study provides strong evidence that both the MRI-based Fagotti score and PCI may serve as accurate, noninvasive predictors of cytoreduction feasibility in ovarian cancer. Both scoring systems showed high discriminatory performance using MRI-derived cut-off values, supporting their potential role in preoperative patient stratification. Their integration into preoperative workflows may assist multidisciplinary teams in selecting patients most likely to benefit from primary debulking surgery. This could result in reducing unnecessary laparotomies and surgical morbidity, optimizing patient stratification for primary debulking versus neoadjuvant chemotherapy, improving individualized treatment planning, and streamlining imaging protocols to enhance clinical applicability. Future multicenter studies should focus on validating these thresholds, assessing inter-institutional reproducibility, and evaluating the real-world clinical impact of MRI-guided surgical triage.

## Figures and Tables

**Table 1:** MRI-based Fagotti score adapted from the laparoscopic index value used to determine peritoneal involvement.

	Score = 0	Score = 2
Peritoneal carcinomatosis	Limited disease (e.g., paracolic gutter, pelvic peritoneum)	Massive peritoneal involvement or miliary disease
Diaphragmatic disease	Isolated diaphragmatic disease	Widespread infiltration or confluent nodules involving most part (>50%) of the diaphragm
Mesenteric disease	Few small nodules	Large infiltrating nodules involving the root of the mesentery or mesenteric retraction
Omental disease	Isolated omental disease	Tumor spread up to the large curvature of the stomach
Bowel infiltration	No infiltration	Extensive bowel involvement or miliary disease
Stomach infiltration	No infiltration	Involvement of the gastric wall
Liver disease	No surface lesions	Any surface lesions

**Table 2:** Clinical, histological and surgical characteristics of the study population.

*n* = 46		*n*	%
**Histological Type**	clear cell	1	2.2
	endometrioid	4	8.7
	high-grade serous	35	76.1
	low-grade serous	4	8.7
	mucinous	2	4.3
**Stage**	Ι	3	6.5
	ΙΙ	3	6.5
	ΙΙΙ	31	67.4
	IV	9	19.6
**Treatment**	NACT	19	41.3
	PDS	27	58.7
**Outcome**	complete	26	56.5
	optimal	1	2.2
	suboptimal/not feasible	19	41.3
		**Mean (SD)**	**Median (IQR)**
**CA-125** **(u/mL)**		1610.5 (1741.4)	723.5 (352–2532)
**MRI-** **based Fagotti**		6 (3.8)	8 (2–8)
**MRI-** **based PCI**		16.7 (7.8)	19.5 (12–23)

**Table 3:** MRI-based Fagotti score and PCI according to surgical outcomes and their results from ROC analysis.

	Resectable Disease										
	No		Yes								
	Mean (SD)	Median (IQR)	Mean (SD)	Median (IQR)	AUC (95% CI)	*p*	Optimal Cut-Off	Sensitivity (%)	Specificity (%)	PPV (%)	NPV (%)
**MRI-based Fagotti**	9.3 (1.8)	8 (8–10)	3.7 (3.2)	4 (0–6)	0.92 (0.84–1.00)	<0.001	≤6	77.8	94.7	95.5	75.0
**MRI-based PCI**	22.9 (2.3)	23 (21–25)	12.4 (7.4)	14 (5–19)	0.94 (0.87–1.00)	<0.001	≤20	92.6	89.5	92.6	89.5

SD: Standard Deviation; IQR: Inter Quartile Range; AUC (95% CI): Area Under the Curve (95% Confidence Interval); PPV: Positive Prognostic Value; NPV: Negative Prognostic Value.

## Data Availability

Imaging data are available at the 1st Department of Radiology, National and Kapodistrian University of Athens, Areteion Hospital. Although these data can be available upon request, they are not uploaded to publicly accessible links due to the General Data Protection Regulation (GDPR) policy of the Hospital.

## Supplementary Materials

The following supporting information can be downloaded at: https://www.mdpi.com/article/10.3390/cancers18010165/s1, Table S1: MRI protocol.

## Abbreviations

The following abbreviations are used in this manuscript:

PCI	Peritoneal Cancer Index
MRI	Magnetic Resonance Imaging
T2W	T2-weighted
DWI	Diffusion Weighted Imaging
T1W	T1-weighted
ROC	Receiver Operating Characteristic
AUC	Area Under the Curve
PDS	Primary Debulking Surgery
IDS	Interval Debulking Surgery
CT	Computed Tomography
PET-CT	Positron Emission Tomography-Computed Tomography
ADC	Apparent Diffusion Coefficient
PIV	Predictive Index Value
SD	Standard Deviation
IQR	Inter Quartile Range
PPV	Positive Predictive Value
NPV	Negative Predictive Value
OR	Odds Ratio
ICC	Intraclass Correlation Coefficient
FIGO	International Federation of Gynecology and Obstetrics
NACT	Neoadjuvant Chemotherapy
CI	Confidence Interval
