# Supplementary material for: Prediction of Resectability of Peritoneal Disease in Ovarian Cancer Patients Using the Peritoneal Cancer Index (PCI) and Fagotti Score on MRI
Source: Cancers (Basel). 2026 Jan 2;18(1):165. doi: 10.3390/cancers18010165 (PMC12785020; doi:10.3390/cancers18010165)
Supplement: Supplementary file 1 [file cancers-18-00165-s001.zip › cancers-4024025-supplementary.pdf]

Table S1. MRI protocol

	Diffusion-weighted (DWI)	T2-WI		T1-WI Pre-contrast	Post-contrast	Post-contrast
Imaging plane	Axial	Axial	Coronal	Axial	Axial	Coronal (pelvis)
Pulse sequence	EPI	MVXD_HR-RT (upper abdomen) mDixon (pelvis)	TSE	mDixon	mDixon	smDixon 3D FFE
Fat suppression	STIR	SPIR (upper abdomen) Dixon (pelvis)	None	Dixon	Dixon	Dixon
b-values (s/mm^2)	0-1000 (upper abdomen) 0-1200 (pelvis)	NA	NA	NA	NA	NA
Repetition time (TR)/ Echo time (TE) (ms)	1900 (upper abdomen) 15900/80 (pelvis)	4100/80 (upper abdomen) 1400/100 (pelvis)	1200/80	3.9/2.4 (upper abdomen) 668/8 (pelvis)	3.9/2.4 (upper abdomen) 711/8 (pelvis)	5.1/2.4
Slice thickness (mm)	5	5	5	5	5	3
Gap (mm)	1	1	1	-2.5 (upper abdomen) 1 (pelvis)	-2.5 (upper abdomen) 1 (pelvis)	-1.5
Number of signal averages (NSA)	1-3 (upper abdomen) 1-9(pelvis)	1	1	1	1	1

EPI: Echo Planar Imaging; MVXD_HR-RT: MultiVane XD_High Resolution; TSE: Turbo Spin Echo; FFE: Fast Field Echo; STIR: Short T1 Inversion Recovery; SPIR: Spectral Presaturation with Inersion Recovery; NA: Not Applicable
